# Therapeutic Value of miRNAs in Coronary Artery Disease

**DOI:** 10.1155/2021/8853748

**Published:** 2021-04-12

**Authors:** Md Sayed Ali Sheikh, A. Alduraywish, A. Almaeen, Mubarak Alruwali, Raed Alruwaili, Basil Mohammed Alomair, Umme Salma, Gomaa Mostafa Hedeab, Najeebullah Bugti, Ibrahim A.M.Abdulhabeeb

**Affiliations:** ^1^Department of Internal Medicine, College of Medicine, Jouf University, Sakaka, Saudi Arabia; ^2^Department of Pathology, College of Medicine, Jouf University, Sakaka, Saudi Arabia; ^3^Department of Gynecology and Obstetrics, College of Medicine, Jouf University, Sakaka, Saudi Arabia; ^4^Department of Pharmacology, College of Medicine, Jouf University, Sakaka, Saudi Arabia; ^5^Pharmacology Department, Faculty of Medicine, Beni-Suef University, Egypt; ^6^Cardiac Center, King Abdul Aziz Specialized Hospital, Sakaka, Saudi Arabia

## Abstract

Atherosclerotic ischemic coronary artery disease (CAD) is a significant community health challenge and the principal cause of morbidity and mortality in both developed and developing countries for all ethnic groups. The progressive chronic coronary atherosclerosis is the main underlying cause of CAD. Although enormous progress occurred in the last three decades in the management of cardiovascular diseases, the prevalence of CAD continues to increase worldwide, indicating the need for discovery of deeper molecular insights of CAD mechanisms, biomarkers, and innovative therapeutic targets. Recently, several research groups established that microRNAs essentially regulate various cardiovascular development and functions, and a deregulated cardiac enriched microRNA profile plays a vital role in the pathogenesis of CAD and its biological aging. Numerous studies established that over- or downregulation of a single miRNA gene by ago-miRNA or anti-miRNA is enough to modify the CAD disease process, significantly prevent age-dependent cardiac cell death, and markedly improve cardiac function. In the light of more recent experimental and clinical evidences, we briefly reviewed and discussed the involvement of miRNAs in CAD and their possible diagnostic/therapeutic values. Moreover, we also focused on the role of miRNAs in the initiation and progression of the atherosclerosis plaque as the strongest risk factor for CAD.

## 1. Introduction

Ischemic coronary artery disease (CAD) is the major public health problem in both developed and developing countries for both genders in all human races. Coronary heart disease (CHD) is responsible for one in seven deaths in the USA, killing over 366,800 people per year. Almost every forty seconds, a USA citizen will have a heart attack, and in the United Kingdom, 1 in 3 men and 1 in 4 women die from acute myocardial infarction (AMI) [1]. Several million patients with chest pain symptoms were usually admitted into high income countries' hospitals each year. About 50% of these patients were diagnosed as CHD, including stable angina, unstable angina, and AMI [1, 2].

Although the mortality rates from CAD in Asian countries are comparatively less than those in the western communities, recently, the number of CAD patients significantly increased due to an increased rate of dyslipidemia, overweight, type-2 diabetes, high blood pressure, and population aging. Around 80% of people who die of CHD are ≥65 years of age. Lifestyle and environmental agents such as unbalanced diets, high consumption of cholesterol-containing fast foods, preserved food with excess sodium chloride, hypovitaminosis D, lack of regular physical exercise, and air pollution are other risk factors [3–5]. Moreover, the prevalence of CAD is expected to increase ~18% by the year 2030 [6]. Accurate identification of risk factors and individuals at risk of developing CAD is the mainstay for CAD prevention. Additionally, early and accurate diagnosis of CAD highly impacts the therapeutic and prognostic outcomes of the disease. Although over the past 30 years significant improvements happened in the management of CAD, it is still necessary to look for newer noninvasive diagnostic and/or therapeutic targets promising to reduce CAD-caused human morbidity and mortality incidence rates.

Accumulating evidence reveals that microinterfering/inhibitory ribonucleic acids (microRNAs, miRNAs, or miRs) could be worth pursuing for new alternative diagnostic and/or treatment strategies for CAD. MicroRNAs are very small noncoding RNAs of about ~22 nucleotide size that regulate target gene expression through posttranscription mechanisms, mainly translational repression and/or mRNA destabilization. It has been accredited that microRNAs are playing essential roles in all human physiological functions including cell cycling, aging, and cellular apoptosis that affect stem cell homeostasis, angiogenesis, and immune function. In this concern, a single miRNA can target several mRNAs. Therefore, the dysregulated biogenesis and homeostasis of miRNAs in tissues have been linked to various diseases, particularly cardiovascular disorders in which they have been examined. The latter include myocardial infarction, remodeling after myocardial infarction, heart failure, dilated cardiomyopathy, cardiac hypertrophy, arrhythmias, atrial fibrillation, valvular heart disease, hypertension, atherosclerosis, and peripheral artery disease. Recently, several clinical research groups acknowledged that miRNAs are detectable in different body fluids including blood plasma and serum. They are extraordinarily protected from degradation by plasma ribonucleases (RNAases) as they are mostly carried out encapsulated within microvesicles [7–11]. The expression of microRNAs was detected in all stages of embryologic cardiac tissue development. For instance, microRNA-29a, microRNA-129, microRNA-21, microRNA-210, microRNA-320, microRNA-423, microRNA-211, and microRNAlet-7c are remarkably expressed in the foetal heart [12]. Several recent studies have reported that the expression of microRNA-27b, microRNA-30d, microRNA-1, microRNA-16, microRNA-126, microRNA-133, microRNA-499, microRNA-143, microRNA-208, and microRNAlet-7 is extremely high in healthy cardiomyocytes and in pathological conditions of the adult heart [13–16]. Moreover, a specific pattern of deregulated circulatory microRNA levels was determined in various forms of ischemic heart disease (IHD). For example, microRNA-208a, microRNA-133a, microRNA-21, and microRNA-126 in stable and unstable CAD [17–20]; microRNA-149, microRNA-765, and microRNA-424 in our previous CAD study [7]; microRNA-1, microRNA-208a, and microRNA-133a in acute coronary syndrome [21]; and microRNA-208b, microRNA-133, and microRNA-1 in myocardial infarction [22]. In addition, circulatory microRNA-21, microRNA-1, microRNA-133a, microRNA-499, microRNA-208a, microRNA-151, and microRNA-331 are invaluable biomarkers for angina pectoris, unstable CAD, and acute heart attack [23–25].

Several excellent studies recognized that miRNAs are critically engaged in various cardiopathophysiological processes and that they have significant diagnostic, prognostic, and therapeutic applications for cardiovascular diseases [8, 26–30]. In this literature review, we largely presented and discussed up-to-dated relevant experimental and clinical evidences on the involvement of various microRNAs in the pathogenesis of CAD and their potential diagnostic, therapeutic, and prognostic applications. Moreover, we also discussed the impact of changes in microRNA expression on the development, progression, and prevention of atherosclerotic plaque formation.

## 2. Literature Searching Methods

We carefully consulted various publications regarding microRNAs and CAD up to January 2021 through searching of different important scientific databases such as microRNA target databases (TargetScan, miRBase, PicTar, and miRDB), Scopus, MEDLINE, PubMed Central, and Embase. CAD and microRNAs, IHD and circulating microRNAs, stable angina and microRNAs, unstable angina and microRNAs, acute coronary syndrome (ACS) and microRNAs, acute myocardial infarction (AMI) and microRNAs, role of microRNAs/miR in atherosclerosis, and therapeutic advantages of microRNAs in CAD were used as keywords during searching the databases. In addition, manual searches were also conducted for related bibliographies from the retrieved articles and very recent review reports.

## 3. Biology of MicroRNA

In 1993, Lee et al. discovered the first miRNA [31], and later, its mode of action was characterized by Wightman et al. [32], while working on the nematode *Caenorhabditis elegans*. They also discovered *lin-4 gene*, which was known to control the timing of *C. elegans* larval development by suppressing the *lin-14* gene. They also discovered two small RNAs that regulate the timing of *C. elegans* development in 2000. Whereas the first-to-second larval stage transition requires lin-4 miRNA, the late larval stage-to-mature nematode transition requires let-7 miRNA. The lin-4 and let-7 miRNAs are homologous to each other, but they are complementary to 3′-untranslated sequences of specific protein-coding genes and negatively controlled the expression-coding genes. Similarly, let-7 miRNA and other small temporally orchestrated miRNAs have the potential to be involved in the regulation of the progressive developmental stages of all organisms including humans [33, 34]. miR-15 and miR-16 located to chromosome 13q14 were first discovered by Calin et al. in 2002. That chromosomal location is known to be deleted in more than 50% of B-cell chronic lymphocytic leukemia (B-CLL). Detailed dissection of that deletion revealed that microRNA-15 and microRNA-16 were situated within a 30 kb area of loss, the region in CLL leading to loss or downregulation of the expression of these two miRNAs in ~68% of CLL. Moreover, these two miRNAs target major players in the cell cycle and apoptosis control such as MCL1, BCL2, ETS1, or JUN.

**Figure 1:**
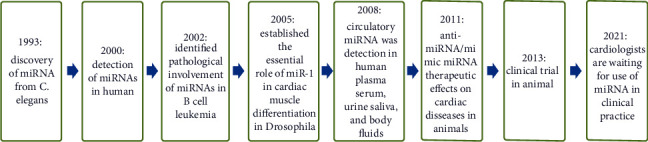
Stages of the discovery history of miRNAs till now.

Recently, miR-15b/16-2 deletion was reported to promote B-cell malignancies by regulating cyclins D1 (CCND1) and D2 (CCND2) and insulin-like growth factor 1 receptor (IGF1R) genes in a mouse model [35–37]. First discovered in 2005, microRNA-1 was found to control cardiomyocyte differentiation and survival through modulating Notch expression and activity [38]. Just one year later, van Rooij et al. reported the first cardiac miRNA profiling and found significant dysregulation afflicting the cardiac remodeling of several miRNAs in both mice and humans [39]. In 2008, Mitchell et al. were first to characterize changes in circulating serum and plasma miRNAs as biomarkers with levels much higher in prostate cancer patients than healthy subjects. Moreover, plasma or serum microRNA levels and profiles correlate each other, and opposite to what is expected for RNAs, they are highly stable against adverse conditions of intense weather such as boiling water, cold temperature, and prolonged storage at room temperature (up to 24 hours). Moreover, these miRNAs are strongly protected from endogenous RNAase activity because they form a protein-miRNA complex in extracellular fluids. In their studies, they used three synthetic microRNA-39, microRNA-238, and microRNA-54 as the internal control as they have no homology with human miRNAs but have significant inhibitory action on RNAase (Figure 1) [40]. Recently, miRNAs were also identified from various body fluids (urine, peritoneal, seminal, saliva, and tears), even in breast milk. They are suggested as novel diagnostic and prognostic biomarkers for cardiac diseases [41].

**Figure 2:**
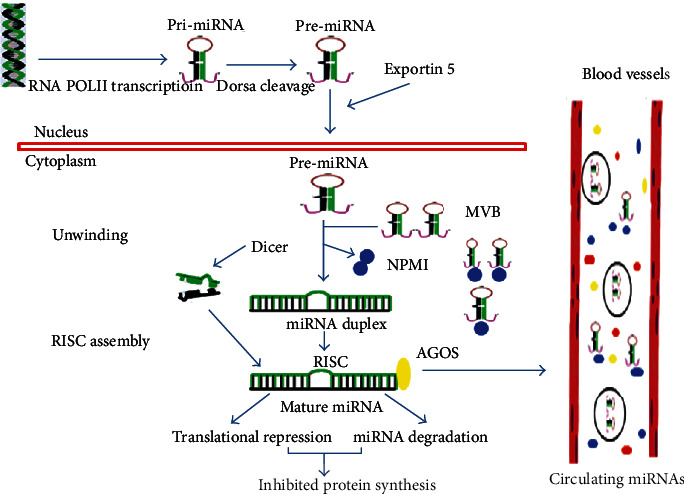
Biology of steps of generating and mechanism of action of circulating miRNAs.

The discovery of microRNA is relatively new. The microRNAs are coexpressed as flanking noncoding parts of the protein-coding initial transcripts of the host genes, expressed from polycistronic genes or intergenic areas or as intronic genomic sequences. Within the nucleus, microRNAs are transcribed by the RNA polymerase II enzyme that forms the primary microRNA transcript of ~2 kb (pri-microRNAs). The latter are cleaved to precursor microRNAs (pre-microRNAs) by RNAase III in association with the help of RNA binding DGCR-8 protein. The pre-microRNAs are small hairclip-shaped RNA molecules of ~100 nucleotides. With introns as the sources, intron splicing generates mirtron pre-microRNAs. Both pre-microRNAs and mirtrons are actively transported into the cytoplasm with the help of GTP-binding nuclear protein and XPO5. Cytoplasmic RNAases recognize mirtrons and pre-microRNA and cut them into short, immature microRNA duplexes (microRNA-microRNA double strand) (Figure 2). Subsequently, the duplex is unwound and converted into mature microRNA by the RISC and protein argonaute. miRNAs work by negatively regulating the gene expression of target genes through translational hindrance and/or hastening mRNA degradation. This is totally dependent on the sequence complementary between the microRNA and the target mRNA. It was believed that passenger fragments and minor miRNAs are functionless and degraded. However, deep sequencing data showed that some of such minor miRNAs are stable and can regulate the homeostasis of other miRNAs as well as modulate the transcription of DNA and translation of RNA. MicroRNA and mRNA interactions usually happened in the Watson–Crick base-pairing manner at the critical seed region located at the 2nd to 8th position of the 5′-end of microRNA to the 3′-UTR end of the targeted mRNA.

Several research groups established that microRNAs are released from different types of cells via cellular exosomes, microparticles/microvesicles, cellular apoptotic bodies, and membrane bounded endocytic vesicles. Both of the healthy myocardium and diseased myocardium release a specific profile of microRNAs which have a potential diagnostic or/and prognostic value for cardiac diseases [8, 11, 15, 42–44].

## 4. MicroRNA Detection Techniques

The real-time PCR arrays are easily accessible, have high efficacy, are nearly available in all the research laboratories, and have been commonly used all over the world for the identification of high-quality microRNA expressions from human, animal, and tissue samples. In addition, nCounter NanoString, microarray, and northern blot techniques are also widely used as an alternative option for quantification of miRNA expressions, but they require larger volumes of RNA and it is sometimes difficult to detect rare types of miRNA.

**Figure 3:**
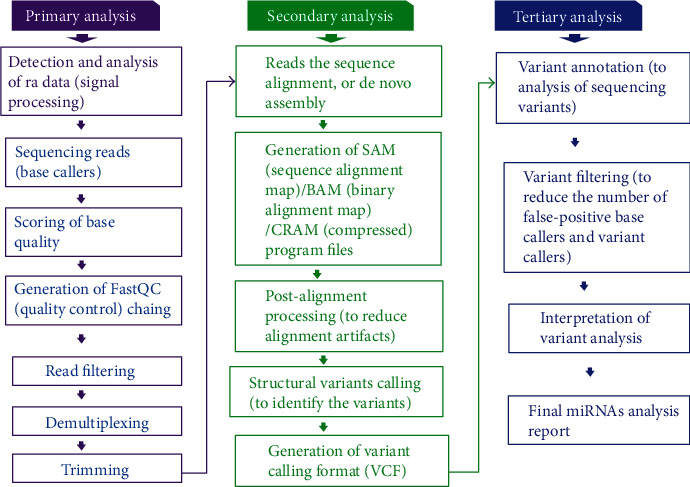
Next-generation sequencing (NGS) analysis.

Currently, next-generation sequencing (NGS) is considered as “the novel and gold-standard” highly reliable technique for the identification of miRNA and is broadly used in clinical laboratory medicine. NGS has the ability to detect the single miRNA, whole genome, and even millions of DNA or RNA sequences simultaneously (Figure 3) [45, 46].

Moreover, NGS is also able to detect unknown miRNAs which the other two generally used miRNA methods (RT-qPCR and microarray) are incapable of doing. Therefore, the NGS technique has been widely used for gene regulation research and profiling of miRNAs in diagnosis, prognosis, and drug development (Table 1) [45, 46].

## 5. Role of miRNAs in the Pathophysiology and Progression of Atherosclerosis and Its Relationship with CAD

### 5.1. Atherosclerotic Plaque Linked with Stable CAD and AMI

Atherosclerosis is a chronic inflammatory disease of the arterial wall caused by endothelial injury and subendothelial accumulation of lipid and extracellular matrix proteins and calcium, particularly at sites of disturbed blood flow that finally form atherosclerotic plaque [47]. Several studies have demonstrated that the plaque is characterized by the accumulation of immune cells, monocytes/macrophages, and T-cells, along with dysfunctional endothelial cells (ECs) with endothelial-to-mesenchymal transition (EndMT) [48]. The formation of an atherosclerotic plaque in the coronary artery is the principal cause for the stable CAD, and the subsequent rupture of the coronary atherosclerotic plaque leading to coronary thrombus is the main reason for unstable angina and AMI.

Accumulating research studies over the last 3 decades recognized that miRNAs play an essential role in regulating pathways controlling lipid homeostasis. Therefore, they are significantly involved in the molecular mechanism of initiation and progression of pathophysiological processes of coronary atherosclerotic plaques. MicroRNAs presented them as promising new diagnostic, therapeutic, and prognostic targets. Normal lipid homeostasis is essential to maintain cellular integrity. Deregulated LDL and HDL metabolism/levels both in the cell and in the circulation are directly linked to various atherosclerotic disease processes. Recent clinical and preclinical studies have identified that microRNAs control LDL and HDL abundance and function and significantly regulate plasma lipoprotein levels. Generation and excretion of various lipoproteins are usually maintained by the hepatocytes. Several liver-associated miRNAs (miR-148a, miR-128-1, miR-130b, miR-122, miR-223, miR-27b, and miR-301b) have been well established to functionally regulate lipoprotein metabolism. Therefore, the altered homeostasis of these miRNAs is critically involved in the production of dyslipidemia [49]. Moreover, hepatic overexpression of miR-30c remarkably decreased atherosclerosis through the downregulation of cholesterol and ApoB-lipoprotein synthesis in a hyperlipidemic mouse model, while inhibition of microRNA-30c induced dyslipidemia and atherosclerosis. Accordingly, upregulation of hepatic microRNA-30c levels might be valuable for the treatment of hyperlipidemias and atherosclerosis [50].

### 5.2. Impact of miRNAs in Coronary Atherosclerosis Progression and Inhibition

**Figure 4:**
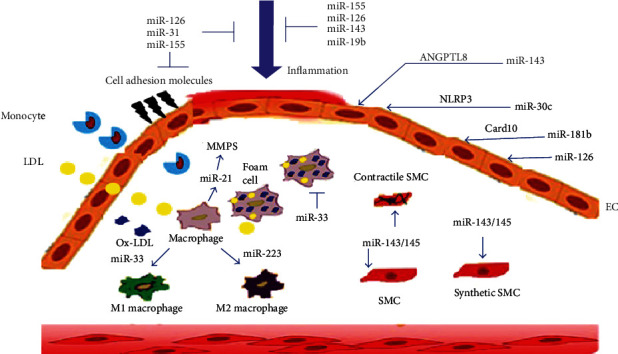
Impact of miRNAs in the pathogenesis of atherosclerosis.

A very recent study noticed that microRNA-30c-5p significantly protected human aortic endothelial cells from oxidized low-density lipoprotein-associated plaque macrophage caspase-1-mediated pyroptotic cell death via downregulation of the NLRP3 protein expression through the forkhead box O3 (FOXO3) pathway. This could be a new treatment approach for atherosclerotic diseases [51]. Angiopoietin-like protein 8 (ANGPTL8) is associated with reduced HDL-cholesterol levels and may contribute to the development of dyslipidemia. Inhibition of miR-143-3p significantly suppressed the ANGPTL8 expression, increased HDL-cholesterol levels, and prevented dyslipidemia and atherosclerosis progression [52]. Administration of microRNA-23a-5p remarkably protected against progression of atherosclerosis and enhanced plaque stability in ApoE−/− mice by decreasing foam cell formation via upregulation of ABCA1/G1 expression levels [53]. Inhibition of miR-33 by anti-miR-33 increased HDL levels and decreased lipid accumulation and inflammation and inhibited the transformation of macrophages into foam cells to markedly reduce atherosclerosis plaque progression [54]. Furthermore, overexpression of miR-155 can significantly inhibit foam cell formation by activating cholesterol ester hydrolase (CEH) in macrophages to decrease intracellular lipid accumulation and prevent atherosclerosis plaque formation [55]. Circulatory levels of microRNA-155 were surprisingly higher in CAD patients as compared with controls. However, anti-miR-155 markedly reduced atherogenesis in ApoE−/− mice by reducing the inflammatory responses of macrophages [56, 57]. The expression of miR-181b is markedly decreased in hyperlipidemic mice and CHD patients than controls. Interestingly, administration of mimic microRNA-181b significantly lowered nuclear factor-*κ*B (NF-*κ*B) activity in endothelial cells and markedly suppressed leukocyte recruitment and reduced atherosclerotic lesion formation in mice [58]. Moreover, overexpression of miR-181b significantly inhibited thrombin-induced endothelial activation and reduced arterial thrombus formation by ~73% via regulating the caspase recruitment domain family member 10 proteins. Besides, microRNA-181b noticeably suppressed NF-*κ*B-linked dependent proteins that include tissue factor, intercellular adhesion molecule-1 (ICAM-1), vascular cell adhesion molecule-1 (VCAM-1), and E-selectin. Therefore, the anti-inflammatory microRNA-181b is a potential target for the prevention of thrombus formation [59]. The involvement of different miRNAs in the pathogenesis of atherosclerosis is illustrated in Figure 4.

Changes in the levels of the endothelium-specific microRNA-126, microRNA-17, and microRNA-92a and the vascular smooth muscle-specific microRNA-145, along with the inflammation-linked microRNA-155, were inversely associated with atherosclerosis plaque formation, while being positively correlated with plaque necrotic and necrolipidic tissue content, and the expression levels were markedly reduced in CAD patients. Therefore, augmentation of these miRNAs by systemic delivery of mimic miRNAs was dramatically reduced by atherosclerosis plaque progression through modulating their target mRNAs [49, 60]. A recent study found that upregulation of microRNA-19b significantly decreased coronary atherosclerotic plaque progression in unstable CAD with the inhibition of endothelial cell activation and new vessel formation through downregulation of the nuclear transcription factor STAT3 [61].

The expressions of miR-21, miR-92a, and miR-99a were significantly upregulated, whereas miR-let-7f, miR-1, and miR-22 were markedly downregulated in human advanced coronary atherosclerotic plaques. Inhibition or overexpression of these miRNAs significantly reduced coronary atherosclerotic plaque progression and rupture [62]. Very recently, Liang et.al reported that miR-124 expressions were decreased in CAD patients and atherosclerotic *ApoE*−/− mouse models, while the mimic expression of miR-124 remarkably reduced anti-inflammatory cytokines and proinflammatory cytokines and inhibited macrophage apoptosis through the suppression of p38, suggesting that overexpression of miR-124 may be considered a promising treatment for atherosclerosis and CAD patients [63]. The antiatherosclerotic mechanisms for miRNAs are summarized in Table 2.

### 5.3. Atherosclerosis Correlation with Statins and miRNAs

Recent reports showed that changes in the expression of microRNAs are closely linked with the therapeutic actions of statins and effectively reduced atherosclerosis plaque formation/rupture through direct or indirect regulation of *β*-hydroxy, *β*-methylglutaryl-CoA (HMG-CoA) reductase, and cytochrome P450 3A and inhibition of proprotein convertase subtilisin/kexin type 9 (PCSK9) to lower LDL uptake via suppressing LDL receptors [64]. Statins inhibit atherosclerosis progression by influencing the integrin signaling pathway. This utilizes the regulation of endothelium-enriched miRNAs (microRNAlet-7e, microRNA-106a, microRNA-342-3p, microRNA-17, microRNA-19a, microRNA-19b, microRNA-24, microRNA-30b, microRNA-30c, microRNA-20a, microRNA-20b, microRNA-93, and microRNA-486-5p) and platelet-specific miRNAs (microRNA-93, microRNA-30c, microRNA-106a, microRNA-425, microRNA-15b, microRNA-17, microRNA-20a, microRNA-20b, microRNA-24, microRNA-19a, microRNA-19b, microRNA-25, and microRNA-484). These miRNAs have antiatherogenic effects in CAD [65]. Though HMG-CoA reductase inhibitors are commonly prescribed drugs for the coronary atherosclerotic heart disease patients, several miRNAs significantly controlled the molecular action of these drugs and also regulated cholesterol homeostasis, endothelial cell inflammation, leukocyte recruitment, formation, stability, and progression of atherosclerosis plaques. As a result, miRNAs may be used as a novel next-generation therapeutic option for the prevention and treatment of atherosclerotic CHD.

## 6. Therapeutic Perspective of miRNAs in CAD

A prospective therapeutic benefit of miRNAs is that they target multiple genes involved in the same pathological pathway process. In contrast, conventional therapies mainly target a single protein, leaving the others unaffected and potentially able to maintain the pathological mechanism. Currently, tools for such a new therapeutic approach utilize microRNA mimics (miR mimics), overexpression, anti-miRNAs (anti-miRs), or inhibitors. In this concern, the anti-miR approach is more appropriate to favor gene suppression, whereas miR mimics are utilized for the upregulation of gene expression. Up to date, the only miRNA-based therapy that reached the clinical application is a Locked Nucleic Acid- (LNA-) modified anti-miR directed against the liver-specific miR-122. The latter is necessary for the replication of hepatitis C virus (HCV) where it binds to two adjacent sequences close to the 5′-end of the HCV genome to guide the RISC-like molecule containing Ago2 binding. Subcutaneous administration of anti-miR-122 at 3-7 mg/kg body weight in chronic hepatitis C genotype 1 virus-infected patients for 5 weeks in a phase II clinical trial showed evidently reduced HCV RNA expression—even in some patients, no HCV RNA was detected up to 14 weeks of follow-up following the treatment. Furthermore, primary safety and the adverse effect profile were excellent. However, the use of anti-miRs in the cardiovascular medicine is still in the preclinical stage [30, 66].

## 7. Therapeutic Role of miRNAs in Stable CAD Patients

A clinical research found that microRNA-34 levels in the plasma of CAD patients were significantly higher than those in non-CAD subjects, whereas the levels of silent information regulator protein-1 (Sirtuin-1, SIRT1 or SIR1) were lower in CAD patients compared with non-CAD subjects. After treatment with the lipid-lowering agent atorvastatin for eight months in CAD patients, the plasma level of miR-34a was significantly reduced while the SIRT1 level was dramatically increased. Therefore, through inhibiting miR-34a, atorvastatin was suggested to play an important protective role for endothelial function in CAD patients [67]. MicroRNA-146a/b and the inflammatory cytokines, such as Toll-like receptor 4 (TLR4), IRAK1, and TRAF6 expressions, are markedly upregulated in CAD patients. On the contrary, a one-year treatment with the hypertensive telmisartan (ARB) and atorvastatin caused significant decreases in microRNA-146a/b and TLR4 levels in CAD patients. Therefore, the inhibitor miR-146a/b may be used in a combination therapy for CAD [68]. In addition, the circulating levels of the TLR4-responsive miRNAs (miR-16, miR-145, miR-31, miR-181a, and miR-let-7i) were significantly lower in patients with CAD than in controls. High levels of these microRNAs are adversely associated with CAD [69, 70].

It is well established that endothelial progenitor cells (EPCs) and vascular endothelial growth factors (VEGF) are essential components for angiogenesis, and circulatory VEGF concentrations are negatively associated with CAD development. Plasma levels of VEGF levels and angiogenic activities were markedly reduced while concentrations of miR-23 and miR-361-5p were significantly elevated in EPCs from CAD patients [71, 72]. On the contrary, knockdown of miR-23 and anti-miR-361-5p evidently restored normal VEGF levels and neoangiogenesis activities of CAD-derived EPCs, but they also significantly improved recovery of blood flow by 90% in the ischemic limbs of mice. This indicates the diagnostic, prognostic, and therapeutic value of variations in plasma levels of microRNA-361-5p and microRNA-23 for CAD patients that are reflected on VEGF [71, 72]. MicroRNA-23a expression is considerably higher in CAD patients and their EPCs. It significantly suppresses epidermal growth factor receptor (EGFR) expression and EPC activities. Conversely, inhibition of microRNA-23a amazingly enhances EGFR expression and new vessel formation by CAD-derived EPCs with an outstanding improvement in blood flow in the hypoxic area. This indicates that miR-23a is a potential therapeutic target for CAD management [73].

Serum levels of each of microRNA-499a-3p and microRNA-135b-5p were 4.7- and 3.9-times, respectively, higher in CAD groups than in normal healthy volunteer subjects. It was also found that the high levels of these two miRNAs could jointly promote ECs and vascular smooth muscle cell (VSMC) proliferation and migration by repressing the myocyte enhancer factor 2C (MEF2C) gene expression. This presents microRNA-499a-3p and microRNA-135b-5p as potential therapeutic targets for atherosclerotic CAD patients [74].

Myogenic miR-502 suppresses the autophagy process and plays an atheropreventive role in CAD by directly targeting the ras oncogene-related GTPase Rab1b and adaptor-related protein complex 2 subunit *β*1 (AP2B1), and downregulation of miR-502 also ameliorates myocardial insult [75]. Rayner et al. reported that inhibition of microRNAs, microRNA-33b and miR-33a, greatly reduced VLDL and highly elevated HDL concentrations through regulating fatty acid oxidation and synthesis through its target genes, carnitine O-octanoyltransferase (CROT), carnitine palmitoyltransferase 1A (CPT1A), fatty acid synthase (FASN), Sterol Regulatory Element Binding Transcription Factor 1 (SREBF1), hydroxyacyl-CoA dehydrogenase trifunctional multienzyme complex subunit *β* (HADHB), ATP-citrate lyase (ACLY), protein kinase AMP-activated catalytic subunit *α*1 (PRKAA1), and acetyl-CoA carboxylase *α* (ACACA). They orchestrate normalization of dyslipidemia and lower CAD risk [76]. The blood level of microRNA-938 was recently shown to be decreased in coronary collateral circulation CAD patients. The ago-miR of microRNA-938 noticeably suppresses EC proliferation and adhesion through its target *γ*-catenin. Moreover, miR-939 controls angiogenesis and is a new treatment option for CAD [77]. Relative to the normal control, the miRNA-155 level was reported to be higher in both CAD patients and atherosclerotic mice. miR-155 prevents the pathogenetic process leading to atherosclerosis and its progression in vivo and in vitro. Therefore, miR-155 is capable of preventing CAD through targeting mitogen-activated protein kinase kinase kinase 10 (MAP3K10) and controlling the inflammatory response and MAPK pathway signaling [78].  Table 3 summarizes the miRNAs involved in CAD and their mechanism of action.

Utilizing several targets, microRNA-126 significantly protects cardiomyocytes against injury. EC-derived apoptotic bodies are produced during atherosclerosis and convey paracrine alarm signals to recipient vascular cells. The latter are induced to produce CXCL12, a process tightly regulated with the EC-enriched microRNA-126. Moreover, systemic delivery of microRNA-126 reduced atherosclerosis plaque, recruited more hematopoietic stem and progenitor cells expressing stem cell antigen 1, and stabilized atherosclerotic plaque in various mouse models. These actions culminate into a significant prevention of atherosclerotic CAD [79]. After balloon injury, miR-21 was aberrantly expressed in the vascular walls. It is also markedly upregulated in atherosclerotic plaques. These effects were reversed after blocking miR-21 actions by antisense oligonucleotide. Moreover, neointima formation was also highly reduced following vascular balloon injury. The latter involves upregulation of the apoptosis-regulating Bcl2 and phosphatase and tensin homologue (PTEN) proteins. These findings suggest that miR-21 is a promising novel regulatory RNA for neointimal lesion formation. It can be exploited in the management of postangioplasty restenosis and atherosclerotic CAD [80].

Establishment of coronary collateral circulation enhances circulation in ischemic cardiac tissue and reduces cardiac mortality. In such a state, levels of circulatory microRNA-146 were noticed to be high in a positive correlation with the extent of coronary collaterals. In addition, miR-146 positively correlates levels of vascular endothelial growth factor A (VEGF-A) with a potential favorable therapeutic effect in CAD patients [81]. In a two-month-old CAD male mouse model, the microRNA-20a level was reported to be decreased. Overexpression of microRNA-20a significantly suppressed thromboxane A2 synthesizing activities, angiotensin II, PTEN, and endothelin-1. Overexpressed microRNA-20a also upregulates VEGF, prostaglandin I2 synthesis, and endothelial nitrous oxide synthetase and improved endothelial cell viability via PI3K/Akt targeting. It also markedly reduced incidence of exercise-associated CAD [82]. The circulating levels of miRNAs, microRNA-106a, microRNA-21, microRNA-126, microR-130a, microRNA-142, and microRNA-27a, were reported to increase during platelet activation and to have a relationship with antiplatelet drugs such as clopidogrel and aspirin. In particular, high levels of microRNA-142 in the plasma were directly associated with major complications of cardiovascular events in atherosclerotic CAD patients after percutaneous coronary intervention (PCI) [83]. It was very recently showed that miR-98 levels were obviously decreased in single-, double-, and multivessel lesion CAD patients and 24-hour hypoxic-induced HUVEC, while ago-miR-98 remarkably suppressed caspase-3 activity, reduced atherosclerosis, and markedly increased cellular viability through targeting LOX-1, which may be used for possible molecular drug targets for atherosclerotic CAD subjects [84].

## 8. Therapeutic Role of miRNAs in AMI Patients

Extensive heart remodeling with fibroblasts happens during myocardium injury that significantly reduces heart function. There are a number of cardiomyocyte-specific microRNAs that regulate the biology of cardiac fibroblasts and its transformation. Adult human cardiac tissue has a very low capacity to regenerate after cardiac tissue injury. The Eulalio research group injected human-microRNA-590 and human-microRNA-199a into myocardial infarction mice. They reported marked enhancement of cardiomyocyte proliferation and an outstanding repair of injured cardiac tissue. This resulted in almost complete recovery of cardiac functional parameters in both neonatal and adult infarcted mice. These two miRs executed their cardiac regeneration therapeutic actions through their target genes, Homer1, homeodomain-only protein X (HOPX), and chloride intracellular channel 5 (Clic5) [85, 86].

MicroRNA-210 is highly expressed in normoxic HL-1 cardiomyocytes of live mice compared with the hypoxic cells. MicroRNA-210 upregulates several angiogenic factors, inhibits caspase activity and apoptosis, significantly improves left ventricular ejection fraction rate, and improves the survival rate in a mouse AMI model. For that, it regulates protein tyrosine phosphatase 1B (PTP1B), hypoxia-inducible factor- (HIF-) 1*α*, and ephrin A3 (EFNA3) genes. These effects nominate miR-210 as a promising novel therapeutic target of AMI [87, 88].

Senescence is a major nonmodifiable risk factor for different types of cardiovascular pathology and contributes significantly bad prognosis in patients with AMI. The aging heart has a high expression rate of miR-34a, and its inhibition significantly reduces the age-associated cardiomyocyte cell death and fibrosis following AMI. This improves the recovery of myocardial function through the regulation of contractile function of the aging cardiac muscle and that of the post-AMI patients. Protein phosphatase 1 regulatory subunit 10 (PPP1R10) is the target gene for miR-34a [89]. Vasculogenesis in ischemic conditions is suppressed in aging people. As compared to human umbilical ECs (HUVECs), the expression of the proangiogenic miR-130a-3p is significantly decreased in aged ECs. Therefore, it was expected that overexpression of microRNA-130a-3p in aged ECs significantly decreased cellular senescence and markedly enhanced the angiogenic activities of ECs. Besides, intramuscular injection of a mimic-microRNA-130a-3p into the ischemic hind limb of old mice restores blood flow and vascular densities in the ischemic tissues with reduced tissue damage. This was accompanied with considerable improvement in functional mobility. Moreover, injection of mimic-microRNA-130a-3p into 15-month-old mice significantly increased bone marrow-derived proangiogenic cell counts following an ischemic insult. These studies revealed the therapeutic potential of mimic-microRNA-130a-3p for severe ischemic vascular diseases [90].

When AMI is induced in neonatal and adult mouse hearts by ligation of coronary artery, the expression of miR-34a was increased 7 days later only in adult but not in neonatal hearts. miR-34a regulates cell cycle and cell death through the modulation of its target genes Bcl2, cyclin D1, and Sirt1. Delivery of an anti-miR-34a to heart tissues diminished such post-AMI miR-34a upregulation and significantly improved post-AMI remodeling [91]. Circulating miR-34a levels in AMI patients and AMI rats were highly increased as compared with healthy controls. Moreover, mimic-miR-34a markedly increased apoptosis through downregulating aldehyde dehydrogenase 2 (ALDH2) gene expression [92]. Overexpression of miR-92a in human ECs suppresses angiogenesis, in vitro and in vivo, and systemic delivery of an anti-ago-miR designed to inhibit miR-92a improved new blood vessel growth and functional recovery of AMI tissues of the mouse model [93]. In a porcine model of ischemia and reperfusion injury, antisense LNA-miR92a significantly reduced the cardiac infarct size compared with controls and improved ejection fraction and left ventricular end-diastolic pressure. Antisense LNA-miR92a was found to have cell-protective, proangiogenic, and anti-inflammatory effects. Therefore, therapeutically, to preserve myocardial and endothelial function after ischemia, miR-92a has to be inhibited [94, 95].

Hullinger et al. demonstrated that systemic administration of the inhibitory microRNA-15 in AMI murine and swine models significantly decreased the cardiomyocyte infarct region, prevented heart remodeling, and improved heart function [96]. Decreases in the level of microRNA-29 in both of mouse and human AMI tissues correlate with increases in collagen expression and fibrosis. On the contrary, overexpression of miR-29 reduces collagen expression and cardiac fibrosis with obviously improved cardiac function [97]. In a mouse AMI model and hypoxic primary cardiomyocytes, microRNA-30 levels are highly increased that correlated with reduced cystathionine-*γ*-lyase (CSE) expression and hydrogen sulfide (H_2_S) production and aggravated hypoxic cardiomyocyte injury. Oppositely, silencing miR-30 prevents the hypoxic cardiomyocyte injury by increasing CSE and H2S levels. Systemic administration of antisense LNA-miR-30 inhibitor increased H_2_S and CSE levels simultaneously with a noticeable decrease in cardiac infarct size and cell death and a markedly enhanced cardiac function in response to AMI [98].

In the aging heart with MI, interstitial cardiac fibrosis is the major cause for deterioration of heart function. After coronary artery ligation in rats, the expression of miR-101a/b in the peri-infarct area is decreased. Overexpression of miR-101a decreased the expression of c-Fos, at protein and mRNA levels. A consequent reduction in transforming growth factor- (TGF-) *β*1 also happened. Increases in miR-101a/b expression reduce collagen production and proliferation of rat neonatal cardiac fibroblasts. Cotransfection with antisense inhibitors of miR-101a/b abrogated these effects. Four weeks after adenovirus-mediated overexpression of miR-101a in rats with chronic MI, there was noticeable improvement in cardiac function [99].

The increases in cardiac endothelial-specific microRNA-24 following acute heart attack is correlated with enhanced apoptosis of cardiac ECs and reduced endothelial new capillary formation. MicroRNA-24 works through modulating the activities of p21-activated kinase 4 (PAK4) and EC transcription factor GATA2. Blocking miR-24 reduces infarct size that showed reduced EC apoptosis and enhanced vascularity with a consequent preservation of cardiac performance and improved survival after AMI in mice [100]. Cardiac repair after MI requires the mobilization of the proangiogenic bone marrow-derived stem/progenitor cells. In this concern, it was found that upregulation of the angiogenic miRs, miR-let-7b and miR-378, is able to enhance the mobilization of the proangiogenic CD34^+^ progenitor cells in patients with STEMI. In particular, miR-378 through controlling CD34^+^ progenitor cells in vivo presents a new endogenous repair mechanism for AMI [101]. In the hypoxic-ischemic neonatal rat cardiomyocytes, microRNA-208 is overexpressed. Oppositely, a treatment with an inhibitor for miR-208 protected hypoxia/ischemia-injured cardiomyocytes through recovering nemo-like kinase (NLK) inhibition [102].

H9c2 cardiomyocytes exposed to hypoxia show high miR-122 expression, and knockdown of microRNA-122 enhances the viability of these cells and reverses the apoptotic effect of hypoxia through modulating the PTEN/PI3K/AKT and cell autophagy pathways [103]. Chun et al. reported a reduction in the level of microRNA-98 in AMI mouse tissue, and overexpression of microRNA-98 prevented cardiac cell apoptosis and lowered blood lactate dehydrogenase levels. These effects were reflected as decreased infarct size and improved cardiac cell viability and cardiac function. MicroRNA-98 works through suppressing the Fas/caspase-3 apoptotic pathway [104]. MicroRNA-21, through inhibiting period circadian protein 2 (PER2), is able to prevent hypoxic cardiomyocyte injury in mouse model of AMI [105]. Human aortic endothelial cells treated with lipopolysaccharides (LPS) to induce plasminogen activator inhibitor-1 (PAI-1) have high levels of microRNA-19. However, an inhibitor to microRNA-19b was able to suppress LPS-induced PAI-1 production. The mechanism used by microRNA-19 to induce PAI-1 is through inhibition of the NAD-dependent deacetylase sirtuin-3. Since microRNA-19 and PAI-1 are implicated in ST-segment elevated MI, miR-19b was proposed as a potential therapeutic venue for AMI [106]. Induction of ischemic reperfusion in mouse heart was followed by increases in the levels of microRNA-125a-5p, microRNA-339-3p, and microRNA-146b expression. Intracardiac nitrite therapy before reperfusion normalized the rate of expression of these miRs. MicroRNA-339-3p and microRNA-146b target the interleukin-1 receptor-associated kinase- (IRAK-) M gene [107].

Li et al. reported a higher expression of microRNA-93 in CAD and AMI patients, a mouse AMI model, and cultured cardiac tissue exposed to hypoxia for 2 hrs. Its role was evidently protective since upregulation of microRNA-93 decreases remodeling with increased vascularity and polarization of the macrophage that caused significant improvement in cardiac activity. On the contrary, knockdown of microRNA-93 significantly aggravated cardiac remodeling. This caused increased left ventricular systolic and diastolic diameter and a reduced ejection fraction rate [108].

miRNA-146 regulates tumor necrosis factor- (TNF-) *α*, interleukin- (IL-) 1, and IL-17 signaling. A recent report demonstrated that miRNA-146 knockdown significantly reduced cardiac apoptosis and improved cardiac function in an AMI rat model [28]. When hypoxia-exposed H9c2 cardiomyocytes and rats with AMI are treated with exosomes derived from miR-146a-modified adipose-derived stem cells, AMI induced myocardial TLR4/NF-*κ*B inflammatory response, apoptosis, and fibrosis. To inhibit TLR4/NF-*κ*B, miR-146a targets mRNA of early growth response factor 1 (EGR1) and prevents its posttranscriptional expression (EGR1) [109]. Researchers noticed the reduction in the levels of expression of miR-133 in the ischemic myocardium. When overexpressed, miR-133 was able to reduce the expression of potassium voltage-gated channel (KCN) subfamily Q member 1 (KCNQ1) and subfamily H member 2 (KCNH2) with reduction in infarct size, improved cardiac function, and suppression of cardiac arrhythmias in AMI mice [29]. It has been demonstrated that elevated expression of miR-328 is a potent determinant of cardiac fibrosis during MI. Therefore, inhibition of miR-328 significantly reduced cardiac fibrosis and improved cardiac function by regulating the TGF-*β*1 pathway [110].

Our recent study demonstrated that miR-375 levels were noticeably downregulated in AMI patients and HR-exposed H9c2 cells, whereas overexpression of miR-375 markedly protected cardiac cell death by inhibiting the expression of caspase-3 via regulating the NLK target, suggesting a potential therapeutic target for AMI patient [111]. The role of different miRNAs in AMI is summarized in Table 4.

## 9. Future Direction and Changeling

The exciting world of miRNA-based research and its potential clinical applications is developing very rapidly. To date, most of the studies dissected the pathogenetic, diagnostic, and prognostic values of miRNAs in cardiovascular diseases. Multicenter larger-scale animal experiments as well as human clinical trials are necessary to establish the therapeutic value of microRNAs as cardiovascular drug targets and/or drugs.

Despite the many reasons to be enthusiastic for miRNAs as a new class of molecular targets or drugs for the treatment of CHD, there are several obstacles that need to be overcome before the clinical application of miRNAs. Although various microRNAs are critically involved in multisystem diseases including CHD, the molecular mechanism of some microRNAs/their mimics and anti-miRs are still largely unknown. Off-target effects for several miRNAs are known. Therefore, further studies on sensitivity, specificity, and potential complications of such effects for cardiac-specific microRNAs must be scrutinized before application.

Since most studies systemically administered these cardiac-specific anti- and mimic miRNAs, they may have secondary effects on other tissues. Therefore, tissue-targeting studies are urgently needed to lower by-standard effects and induce efficiency. Additionally, larger scale multicenter animal studies are needed to confirm the efficacy, half-life, and reversibility of anti-/mimic microRNAs. The detection of miRNAs by the use of qRT-PCR or droplet digital PCR (ddPCR) techniques is time consuming, expensive, and even gives some false-positive reports. Therefore, it is necessary to develop faster and cost-effective modified PCR machines.

However, currently, most of the advanced clinical laboratories and highly sensitive and specific, more accurate, comparatively cheaper and less time consuming, enormous potential, and breakthrough NGS techniques have been used for the detection of miRNAs as clinical biomarkers for diagnosis, prognosis, and potential pharmaceutical targets for the management of CAD patients [46, 112]. Hopefully, within the coming few years, mimic and/or inhibitory miRNAs will be used as a promising novel therapeutic strategy for the treatment of cardiovascular disease patients.

## Figures and Tables

**Table 1:** Updated commonly used next-generation sequencing (NGS) platforms.

Company name with platforms	Launched year	Applications/sequencing mechanism	Maximum read length (nucleotide)
Roche/454, GS FLX (genome sequencing)	2005	Pyrosequencing	~1,000 bp
Illumina, MiSeq, HiSeq 2500, HiSeq 4000, Novate	2006	Reversible terminator	~300 bp
Helicos BioSciences, Single Molecule Sequencing (SMS)	2009	Fluorescent	~200 nt
PacBio, Sequel	2011	Real time	~15 kb
Ion Torrent/Thermo Fisher, PGM (Personal Genome Machine)	2012	Detection of hydrogen ion	~400 bp
Oxford Nanopore, MinION/GridION	2015	Real time	>2 Mb
SeqLL, “True Single Molecule Sequencing (tSMS)”	2017	Helicos sequencing/HeliScope single-molecule sequencers	~1,500 bp

**Table 2:** Therapeutic role of miRNAs in atherosclerosis.

miRNAs	Effects and targets	References
Mimic-miR-30c	Reduces hyperlipidemia and atherosclerosis development through decreasing lipid synthesis and apolipoprotein B (APOB) secretion by inhibiting microsomal transfer protein (MTP) activity in ApoE−/− mice	[50]
Mimic-microRNA-30c-5p	Markedly reduces atherosclerosis in human aortic endothelial cells (HAECs) via downregulation of the inflammatory NLRP3 protein expression and forkhead box O3 (FOXO3)	[51]
Anti-miR-143-3p	Increases HDL-cholesterol levels and prevents dyslipidemia and atherosclerosis progression through angiopoietin-like protein 8 (ANGPTL8) in liver cells	[52]
Anti-microRNA-23a-5p	Significantly protects against atherosclerosis and enhances plaque stability in ApoE−/− mice via upregulation of cholesterol transporter ABCA1/G1	[53]
Anti-miR-33	Increases HDL levels, enhances cholesterol efflux, inhibits inflammation and the transformation of macrophages into foam cells, and reduces atherosclerosis plaque progression in ApoE−/− mice	[54]
Anti-miR-155	Reduces atherosclerosis lesion formation in ApoE−/− mice by reducing inflammatory responses of macrophages and enhances cholesterol efflux through targeting suppressor of cytokine signaling 1 (SOCS-1)	[57]
Mimic-miR-181b	Decreases endothelial nuclear factor-*κ*B (NF-*κ*B) activation, suppresses leukocyte recruitment, and prominently declines atherosclerosis in mice through modulating its target gene, importin-*α*3	[58]
Mimic-miR-181b	Suppresses NF-*κ*B-linked proteins that include tissue factor, vascular cell adhesion molecule-1, E-selectin, and intercellular adhesion molecule-1 and decreases arterial thrombus formation by ~73% via caspase recruitment domain family member 10 (Card10) proteins	[59]
Mimic-miR-181b	Delays unstable plaque progression in patients with unstable coronary artery disease by inhibiting endothelial cell proliferation and migration and angiogenesis through STAT3 transcriptional activity in EA.hy926 cells	[61]

**Table 3:** Role of miRNAs in coronary artery disease (CAD).

miRNAs	Target gene(s)	Inhibitory/activating	Actions	References
miR-34a	Silent information regulator protein-1 (SIRT1)	Inhibition	Protects endothelial function in CAD patients	[67]
miR-361-5p	Vascular endothelial growth factor (VEGF)	Inhibition	Induces ~90% improvement in local circulation of the ischemic mouse limbs	[71]
miR-23a	Epidermal growth factor receptor (EGFR)	Inhibition	Improves blood flow recovery in ischemic area	[73]
miR-135b, miR-499a	Myocyte enhancer factor 2C (MEF2C)	Overexpression	Promotes endothelial cells and vascular smooth muscle cell proliferation	[74]
miR-502	GTPase Rab1b and adaptor-related protein complex 2 subunit *β*1 (AP2B1)	Inhibition	Suppresses autophagy	[75]
miR-33a and miR-33b	Carnitine O-octanoyltransferase (CROT), carnitine palmitoyltransferase 1A (CPT1A), hydroxyacyl-CoA dehydrogenase trifunctional multienzyme complex subunit *β* (HADHB), and protein kinase AMP-activated catalytic subunit *α*1 (PRKAA1)	Inhibition	Reduces VLDL and elevates HDL concentrations	[76]
miR-939	*γ*-Catenin	Overexpression	Remarkable role in angiogenesis	[77]
miR-155	Mitogen-activated protein kinase kinase kinase 10 (MAP3K10)	Inhibition	Prevents the development and progression of atherosclerosis and CAD	[78]
miR-126	C-X-C motif chemokine ligand 12 (CXCL12)	Overexpression	Significantly prevents atherosclerotic CAD	[79]
miR-21	Apoptosis-regulating proteins Bcl2 and phosphatase and tensin homologue (PTEN)	Inhibition	Prevents neointimal lesion formation and postangioplasty restenosis	[80]
miR-146	Vascular endothelial growth factor A (VEGF-A)	Overexpression	Increases coronary collateral circulation in ischemic cardiac tissue	[81]
miR-20	Endothelin 1 (ET-1), thromboxane A2 (TXA2) synthesizing enzymes, angiotensin II (ANGII), PTEN	Overexpression	Markedly reduces the incidence of exercise-associated CAD	[82]

**Table 4:** Role of miRNAs in AMI.

miRNAs	Target gene(s)	Inhibitory/activating	Actions	References
hsa-miR-590 and hsa-miR-199a	Homeodomain-only protein homeobox (HOPX), Homer1, and chloride intracellular channel 5 (Clic5)	Activation	Enhances cardiac regeneration and recovery of cardiac function after MI	[85, 86]
miR-210	Hypoxia-induced factor- (HIF-) 1*α*, ephrin A3 (EFNA3), and protein tyrosine phosphatase 1B (PTP1B)	Inhibition	Inhibits caspase activity and apoptosis and upregulates angiogenic factors	[88]
miR-34a	Bcl2, cyclin D1, protein phosphatase 1 regulatory subunit 10 (PPP1R10), and Sirt1	Inhibition	Reduces age-associated cardiomyocyte cell death and fibrosis	[89, 91]
miR-130a-3p	Bone marrow-derived proangiogenic cells	Activation	Increases circulation in ischemic tissue area	[90]
miR-92a	CD31+/CD42b-	Inhibition	Enhances new blood vessel growth and functional recovery of ischemic damaged tissue and reduces cardiac infarct size	[93–95]
miR-30	Cystathionine-*γ*-lyase (CSE) and H_2_S production	Inhibition	Reduces infarct size and apoptotic cell number and enhances cardiac function in response to AMI	[98]
miR-101a	Transforming growth factor- (TGF-) *β*1	Activation	Suppresses cardiac fibroblast proliferation and collagen production and improves cardiac function in chronic MI of rats	[99]
miR-24	GATA2 and p21-activated kinase (PAK) 4	Inhibition	Reduces myocardial infarct size and enhances vascularization	[100]
miR-378	CD34^+^ progenitor cells	Activation	Proangiogenic activity and endogenous repair mechanism	[101]
miR-208	Nemo-like kinase (NLK)	Inhibition	Protects hypoxic/ischemic cardiomyocytes from injury	[102]
miR-98	Fas/caspase-3 apoptotic signal pathway	Activation	Decreases infarct size and increases cardiac cell viability with improved cardiac function	[104]
miRNA-146	Tumor necrosis factor- (TNF-) *α*, interleukin-1 (IL-1), early growth response factor 1 (EGR1), and interleukin-17 (IL-17)	Inhibition	Reduces cardiac apoptosis and improves cardiac function in AMI model	[28, 109]
miR-133	Potassium voltage-gated channel (KCN) subfamily Q member 1 (KCNQ1) and subfamily H member 2 (KCNH2)	Overexpression	Enhances heart function and reduces cardiac arrhythmias in MI	[29]
miR-328	TGF-*β*1 pathway	Inhibition	Reduces cardiac fibrosis and improves cardiac function	[101, 110]
